# Dietary ceramide 2-aminoethylphosphonate, a marine sphingophosphonolipid, improves skin barrier function in hairless mice

**DOI:** 10.1038/s41598-020-70888-0

**Published:** 2020-08-17

**Authors:** Nami Tomonaga, Yuki Manabe, Kazuhiko Aida, Tatsuya Sugawara

**Affiliations:** 1 Laboratory of Technology of Marine Bioproducts, Division of Applied Biosciences, Graduate School of Agriculture, Kyoto University, Kitashirakawaoiwakecho, Sakyo-ku, Kyoto, 606-8502 Japan; 2 Innovation Center, Nippon Flour Mills Co., Ltd, 5-1-3 Midorigaoka, Atsugi, Kanagawa 243-0041 Japan

**Keywords:** Nutrition, Lipids

## Abstract

Sphingolipids are one of the major components of cell membranes and are ubiquitous in eukaryotic organisms. Ceramide 2-aminoethylphosphonate (CAEP) of marine origin is a unique and abundant sphingophosphonolipid with a C-P bond. Although molluscs such as squids and bivalves, containing CAEP, are consumed globally, the dietary efficacy of CAEP is not understood. We investigated the efficacy of marine sphingophosphonolipids by studying the effect of dietary CAEP on the improvement of the skin barrier function in hairless mice fed a diet that induces severely dry-skin condition. The disrupted skin barrier functions such as an increase in the transepidermal water loss (TEWL), a decrease in the skin hydration index, and epidermal hyperplasia were restored by CEAP dietary supplementation. Correspondingly, dietary CAEP significantly increased the content of covalently bound ω-hydroxyceramide, and the expression of its biosynthesis-related genes in the skin. These effects of dietary CAEP mimic those of dietary plant glucosylceramide. The novel observations from this study show an enhancement in the skin barrier function by dietary CAEP and the effects could be contributed by the upregulation of covalently bound ω-hydroxyceramide synthesis in the skin.

## Introduction

The mammalian skin barrier is in the stratum corneum, the outermost layers of the epidermis, which protects against excessive transepidermal water loss (TEWL) and to block of irritants. In this study, we focused on the function to retain water in the epidermis as the skin barrier and the epidermal structures which conducive to the barrier. Lipid lamellae in the extracellular space of corneocytes play a vital role in the barrier function and maintain a hydrophobic environment. These lipids, consisting of 50% ceramides, 25% cholesterol, and 15% fatty acids (on a total lipid mass basis), contribute to the water-holding properties and prevent desiccation by TEWL^1,3^. Ceramide formation occurs by binding of a fatty acid to an amide group of the sphingoid base. The molecular structures of ceramides are various. Ceramides are essential for the skin barrier function since changes in ceramide profile of the lipid lamellae have been associated with impaired barrier function^4–10^.

The structure formed by the binding of ω-hydroxyceramides to cornified envelope proteins is important for the skin barrier^11,12^. The cornified envelope is a rigid structure with an outer lipid layer and an inner protein, which is produced by the crosslinking of precursor proteins such as involucrin and loricrin^13^. Ultra-long-chain ceramide participates in the formation of covalently bound ω-hydroxyceramides^14,15^. The amount of covalently bound ω-hydroxyceramides correlates with skin hydration and skin barrier function^11,12,16^.

Sphingoid base is a common structure of sphingolipids which are one of the major families of lipids. Since sphingolipids are components of cell membranes, they are ubiquitous in eukaryotic organisms^17^. However, the polar head groups and ceramide structure of sphingolipids vary among biological species^17^. For example, sphingomyelin, which has a phosphocholine as a polar head, is a major mammalian sphingophospholipid, and is present in foods such as meat and milk. Glucosylceramide (GluCer) has a glucose as a head group and is a major glycosphingolipid frequently found in not only animals but also higher plants (cereals, beans, and vegetables). A certain quantity of sphingolipids is ingested daily from meals^18,23^. Recent reports show a protective effect on the skin barrier function by dietary intake of sphingomyelin and GluCer^16,24,27^. Dietary sphingomyelin and GluCer enhanced mRNA expression of epidermal ceramide synthases (CERS), contributing to ultra-long-chain ceramide synthesis in the dry-skin hairless mouse model^26^. Sphingoid bases from dietary sphingolipids might participate in the upregulation of epidermal ultra-long-chain ceramide synthesis because sphingoid bases increase the expression of these CERS genes in normal human foreskin keratinocytes^26^. Earlier studies reported that dietary GluCer is digested intestinally and absorbed as sphingoid bases into the lymph^27,29^. Additionally, dietary milk phospholipids (consisted mainly of phosphatidylcholine and sphingomyelin) increased epidermal covalently bound ω-hydroxyceramides, and improved skin barrier function in hairless mice^16^.

In contrast, general marine sphingolipid, ceramide 2-aminoethylphosphonate (CAEP), frequently contains unique structures^30^. Unlike the C-O-P linkage encountered in the polar head of major sphingophospholipids such as sphingomyelin, the phosphorus atom of 2-aminoethylphosphonate, the polar head of CAEP is directly bound to a carbon atom (C-P bond)^30,31^. Sphingolipids with C-P bonds, including CAEP, are sphingophosphonolipids^30^. CAEP consists of not only sphingosine (d18:1) and hexadeca-4-sphingenine (d16:1), often found in mammals, but also a unique triene-type, odd-numbered carbon chain sphingoid base, 2-amino-9-methyl-4,8,10-octadecatriene-1,3-diol (d19:3), which differ from those in mammals^32–37^. The nomenclature for sphingoid bases is to indicate the number of hydroxyl groups (d for di- and t for tri-) followed by the chain length and number of double bonds. CAEP is widely present in marine invertebrates^38^, including molluscs such as squids^39^ and bivalves^40^, consumed globally. Previously, we showed digestion of dietary CAEP to sphingoid bases and absorption of these sphingoid bases, including unique d19:3 into the lymph in experimental animal models^41,42^. Therefore, dietary CAEP has the potential to improve the skin barrier function via modulation of ceramide synthesis. In the present study, we evaluated the effect of dietary CAEP in comparison with GluCer on the skin barrier function to elucidate its mechanism of action by focusing on the synthesis of covalently bound ω-hydroxyceramides.

## Results

### Effect on skin properties

**Figure 1:**
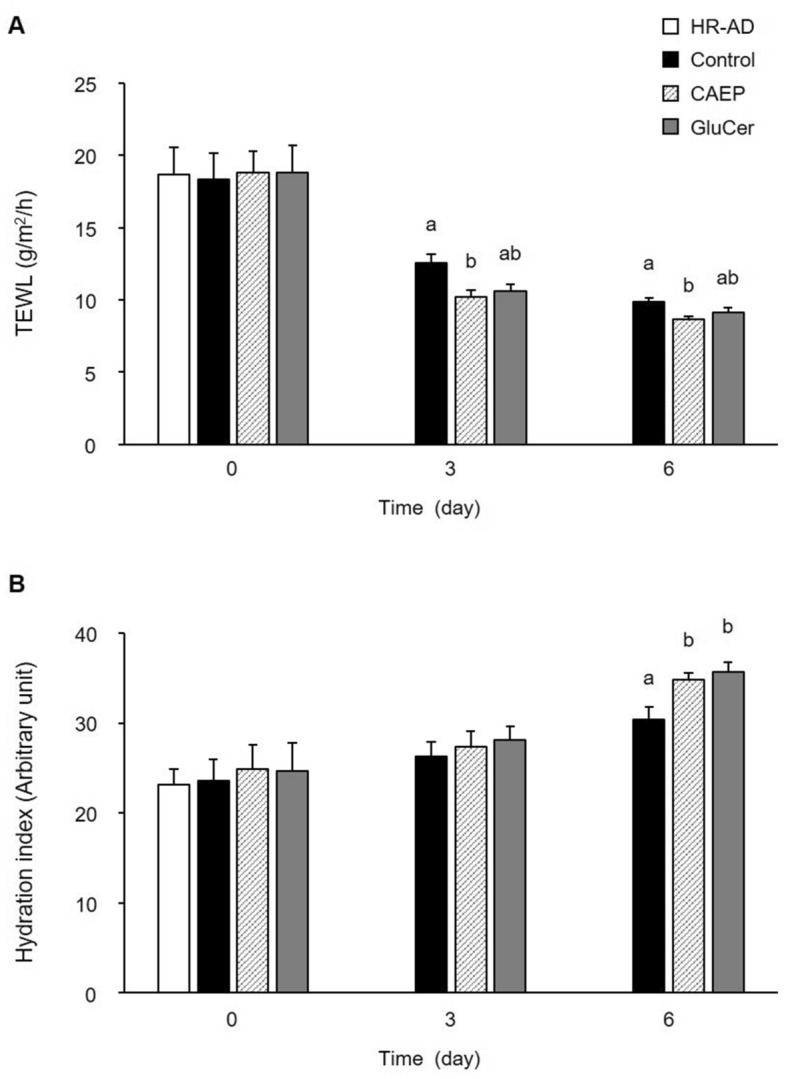
Effect of dietary sphingolipids on TEWL levels (**A**) and the hydration index (**B**) in the HR-AD induced barrier perturbation model. The dorsal skin measurements performed every three days during the recovery period. Data reported as means ± standard errors (HR-AD and control groups, n = 5; CAEP and GluCer groups, n = 6). Bars with different letters at each time point are significantly different from each other by one-way ANOVA, followed by Tukey–Kramer tests (*P* < 0.05).

After feeding mice HR-AD diet for 11 weeks, severe dry-skin and systemic erythema manifested. A HR-AD diet is used to induce skin damages with dry-skin condition and is especially characterized by a deficiency of magnesium^43,45^. The dry-skin showed an increase in the TEWL to 18.7 ± 0.9 g/m^2^/h with a decrease in the hydration index to 24.2 ± 1.3 (arbitrary unit) at the end of HR-AD diet feeding period. The body weight of HR-AD group at the end of HR-AD diet feeding period (day 0 of the recovery treatment period) was 23.1 ± 1.0 g. Daily food intake and body weight were not significantly different among each group during the recovery treatment period (data not shown). After feeding the experimental diet for seven days, tissue weights (liver, spleen, and kidney) were not significantly different among different groups (data not shown). During the recovery treatment period, the dry-skin improved dramatically in all groups. Compared to the control group, TEWL levels on day 3 and 6 of recovery treatment period were reduced significantly by dietary CAEP (Fig. 1A). The hydration index in CAEP and GluCer groups were significantly increased compared to the control group on day 6 of the recovery treatment period (Fig. 1B).

**Figure 2:**
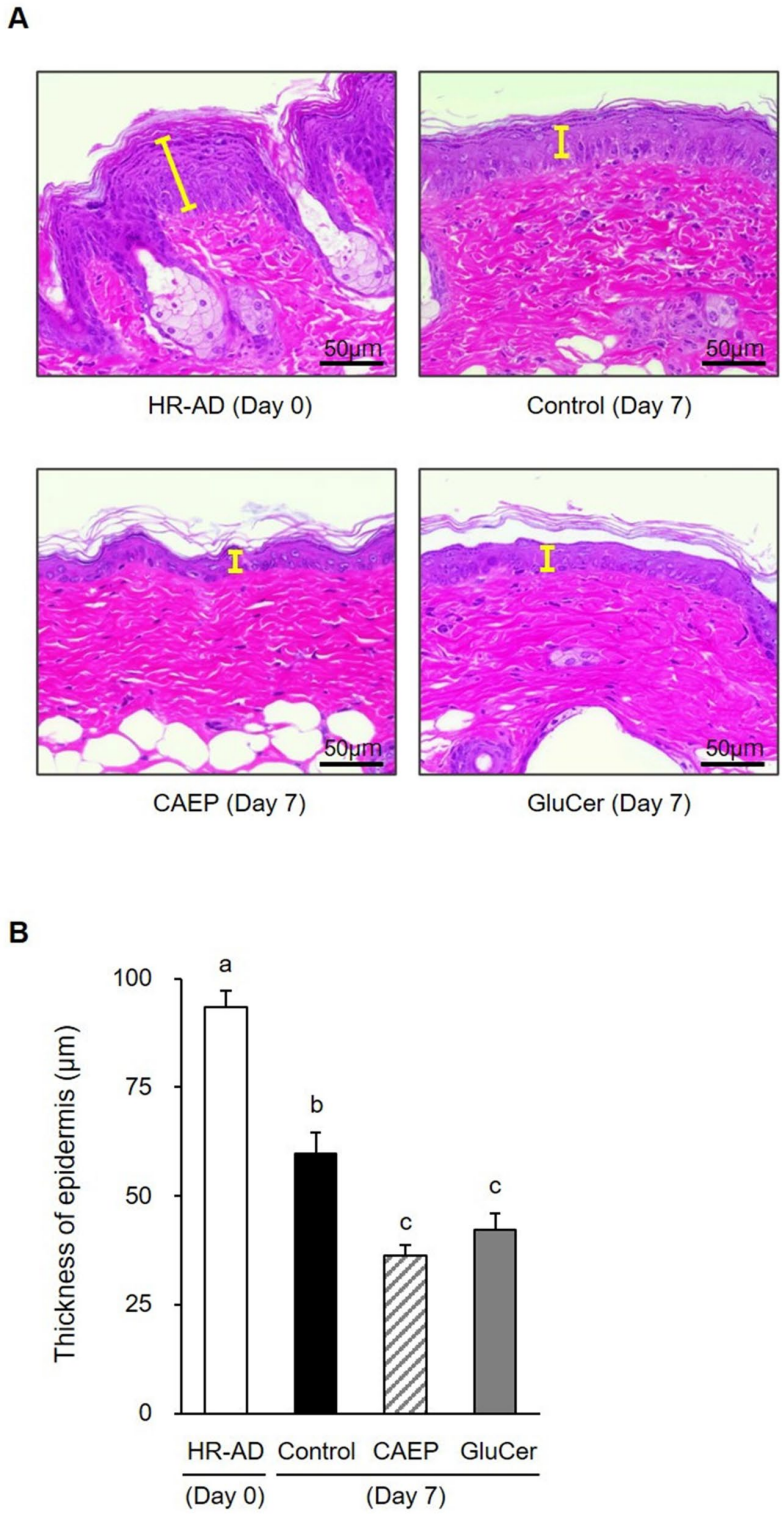
The thickness of the epidermis in mice fed different diets (HR-AD and control groups, n = 5; CAEP and GluCer groups, n = 6). Photographs of mice dorsal skin sections stained with H&E (**A**). The thickness of each specimen was measured using a microscope (**B**). Values reported as means ± standard error. Data analysed by one-way ANOVA, followed by Tukey–Kramer tests. Bars with different letters are significantly different with *P* < 0.05.

**Figure 3:**
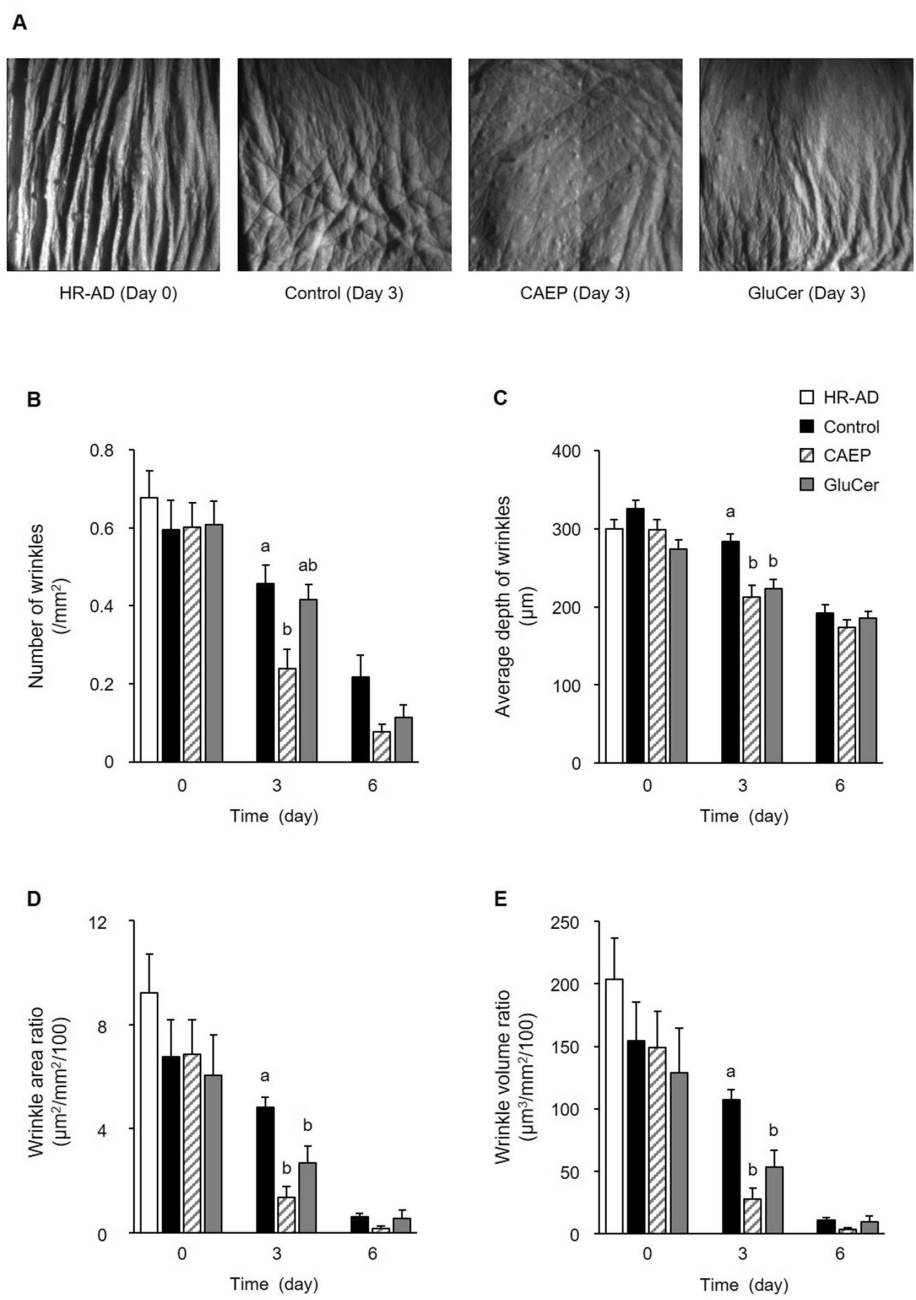
Representative photographs of replicas taken from mice dorsal skin (**A**). The images show skin wrinkles in each group. The number (**B**), the average depth of wrinkles (**C**), wrinkle area ratio (**D**), and volume ratio (**E**) analysed by an imaging analyser. Data reported as means ± standard errors. Bars with different letters at each time point are significantly different from each other by one-way ANOVA, followed by Tukey–Kramer tests (*P* < 0.05).

Epidermal hyperplasia was caused by disruption of the skin barrier in mice fed HR-AD diet for 11 weeks. CEAP and GluCer treatment reduced the thickness of epidermal hyperplasia and improved the skin appearance compared to control mice (Fig. 2). Deep wrinkles developed by feeding the HR-AD diet (Fig. 3A). In comparison with the control group, dietary CAEP significantly decreased the number of wrinkles by day 3 of the recovery period (Fig. 3B). In addition, the average depth of wrinkles, wrinkle area ratio, and wrinkle volume ratio were reduced significantly by ingestion of CAEP and GluCer compared to control diet on day 3 of the recovery period (Fig. 3C – E). These results showed accelerated recovery of the skin barrier function and condition by dietary CAEP, in the dry-skin induced by HR-AD diets, comparable to dietary GluCer.

**Figure 4:**
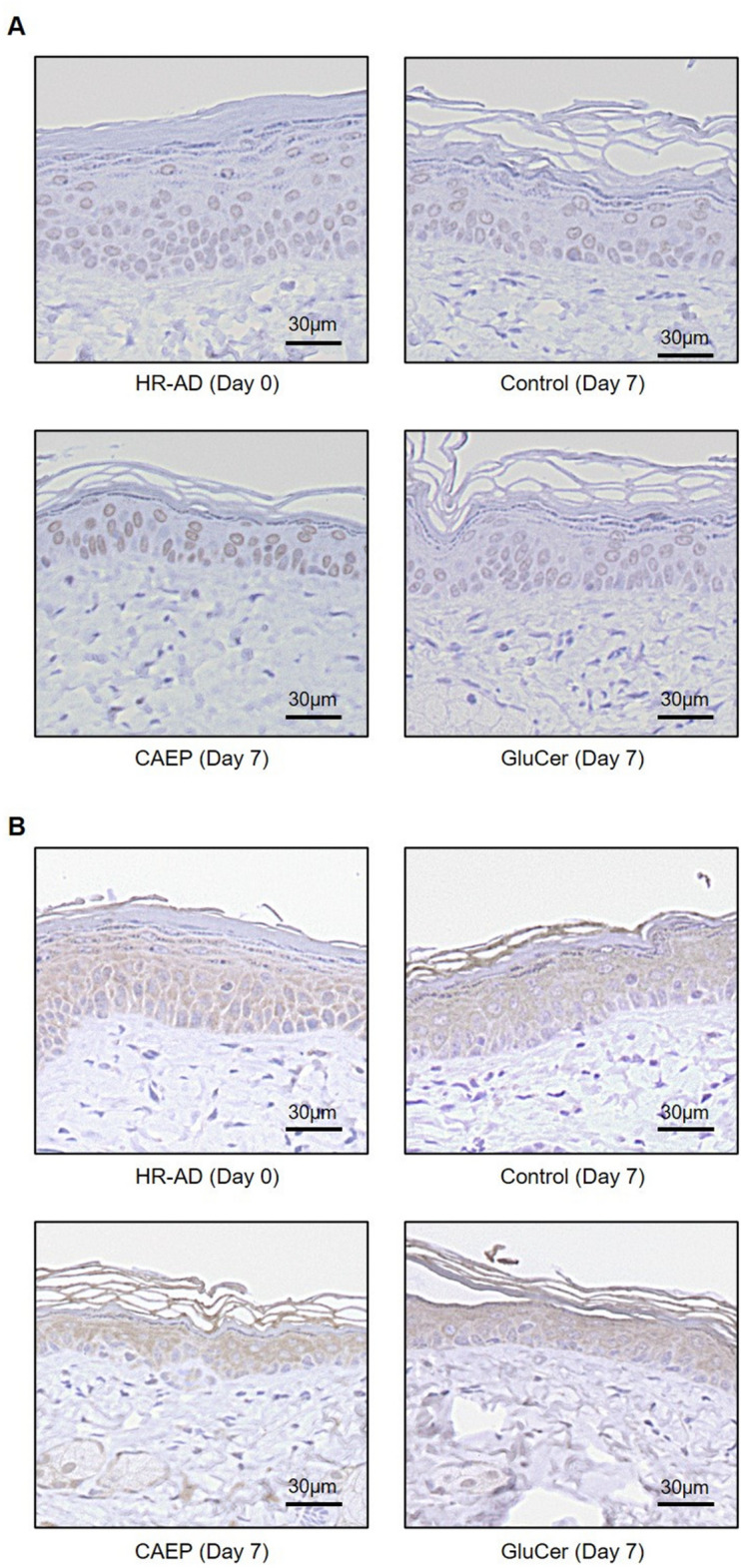
Immunohistochemistry of mice dorsal skin after fed different diets. Photographs of immunohistochemically stained sections with an antibody against involucrin (**A**) and filaggrin (**B**).

Immunohistochemical staining of skin sections showed involucrin localized to keratinocytes (Fig. 4A). In semi-quantitative assay using immunostaining pictures, the average of percentage areas positive for involucrin immunostaining in adequate area (800–900μm^2^) of epidermis were 4.9% for HR-AD group, 8.5% for Control group, 16.9% for CAEP group, and 11.6% for GluCer group (n = 2). In CAEP and GluCer groups, the number of keratinocytes was comparable to the control group. In the stratum corneum, the staining intensity of filaggrin was not different among control, CAEP, and GluCer groups (Fig. 4B).

### Effect on covalently bound ω-hydroxyceramide synthesis in the skin

**Figure 5:**
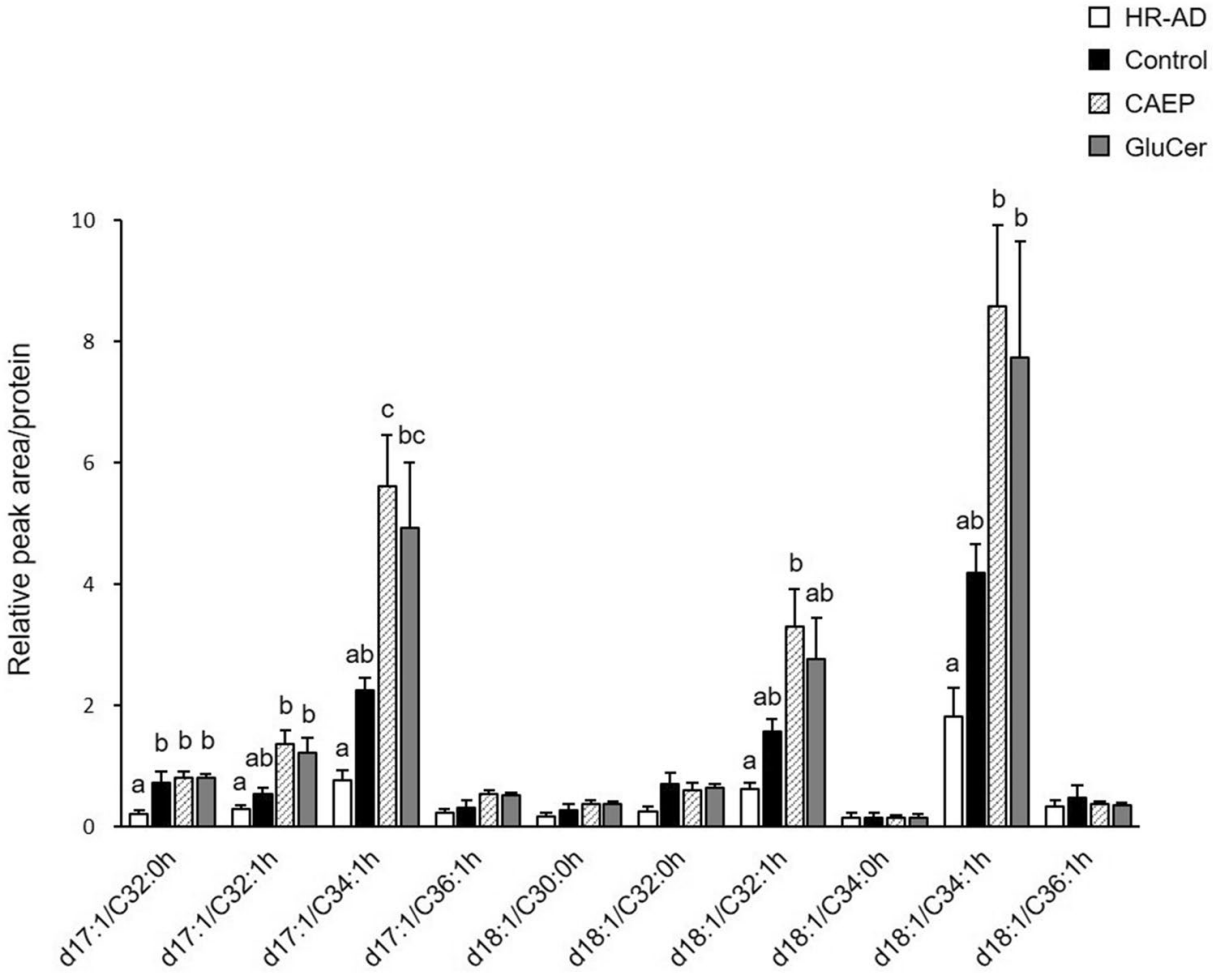
Levels of covalently bound ω-hydroxyceramides in mice epidermis fed different diets (HR-AD and control groups, n = 5; CAEP and GluCer groups, n = 6). The relative peak area per epidermal protein content presented as the fold change relative to ω-hydroxyceramide standard (d18:1/30:0 h, 1.0 pmol). Data reported as means ± standard errors and were analysed by one-way ANOVA, followed by Tukey–Kramer tests. Bars with different letters in each molecular species are significantly different with *P* < 0.05.

Each ceramide molecules structure is shown as “sphingoid base/fatty acid” (“h” placed at numerical symbols of fatty acid means hydroxyl group) with reference to the nomenclature^46^. The covalently bound ω-hydroxyceramides levels were elevated during the recovery treatment period (Table S1, Fig. 5). Especially, d17:1/32:0 h, d17:1/32:1 h, d17:1/34:1 h, d18:1/32:1 h, and d18:1/34:1 h increased significantly by dietary CAEP and GluCer compared with the HR-AD group. These ω-hydroxyceramide molecules excluding d17:1/32:0 h increased in CAEP and GluCer groups, and d17:1/34:1 h showed a particularly significant change in CAEP group compared with the control group.

**Figure 6:**
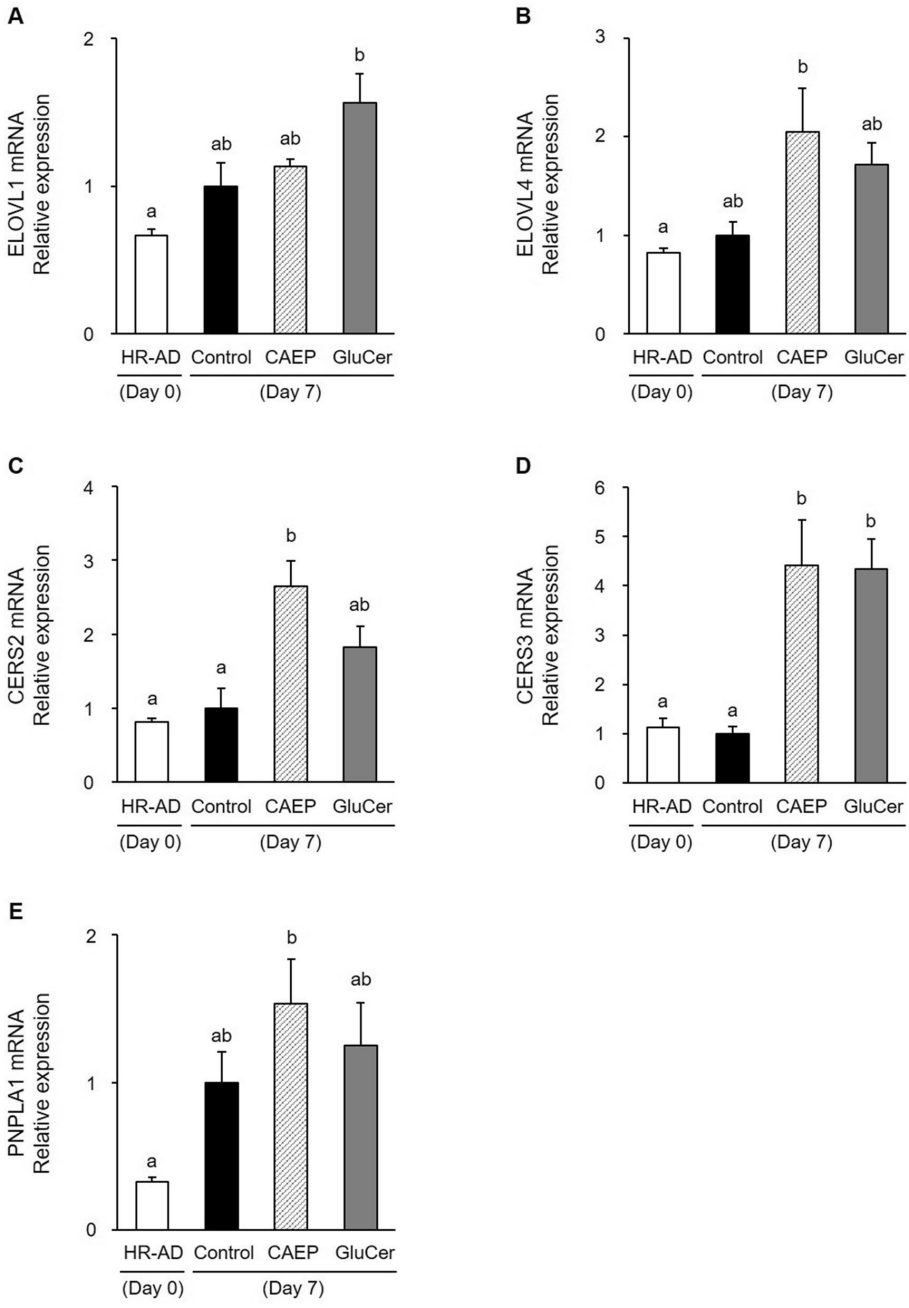
Effect of dietary sphingolipids on the expression of genes related to the synthesis of covalently bound ω-hydroxyceramides in the dorsal epidermis of the AD-like murine model. The quantification of relative expression of ELOVL1 (**A**), ELOVL4 (**B**), CERS2 (**C**), CERS3 (**D**), and PNPLA1 (**E**) mRNAs was by real-time RT-PCR. The expression of ACTB mRNA was used as an internal control. Values presented as means ± standard errors (HR-AD and control groups, n = 5; CAEP and GluCer groups, n = 6). Data analysed by one-way ANOVA, followed by Tukey–Kramer tests. Bars with different letters are significantly different with *P* < 0.05.

Multiple enzymes contribute to the synthesis of covalently bound ω-hydroxyceramide. In this study, dietary CAEP increased mRNA expression of fatty acid elongase (ELOVL4) and ceramide synthases (CERS2 and 3) in the dorsal skin of mice (Fig. 6B–D), especially CERS2 and 3 mRNA expression levels in CAEP group increased significantly than in the HR-AD and control groups (Fig. 6C and D). Additionally, patatin-like phospholipase domain-containing protein 1 (PNPLA1) mRNA expression in CAEP group was significantly higher compared with the HR-AD group (Fig. 6E). PNPLA1 catalyses the synthesis of acylceramide, which is a precursor of covalently bound ω-hydroxyceramide^15,47,48^. Dietary GluCer significantly upregulated mRNA expression of ELOVL1 and CERS3 in the skin, compared with the HR-AD diet (Fig. 6A and D). These results suggested that dietary CAEP and GluCer promoted the synthesis of covalently bound ω-hydroxyceramides.

## Discussion

Our results show an improvement of skin barrier function by dietary CAEP, and these effects were similar to that of dietary GluCer. The content of CAEP from squids is about 0.2–2% (weight percent of total lipids)^39^, thus supplementation of CAEP would be useful to achieve the functions. In the present study, the dry-skin condition was produced by feeding HR-AD diet as described earlier^47,48^. Dietary CAEP enhanced the reduction of TEWL and the increment of the skin hydration index during the recovery treatment period in this model. The epidermal hyperplasia is associated with skin dryness and indicated by the disordered skin barrier^49,50^. The epidermal thickness in CAEP group from this study closely matches that in normal mature hairless mice HR-1 (approximately 10–15 μm ^43^). Wrinkles frequently appear due to the dry-skin, such as photodamaged and aged skin, which is closely related to the skin barrier disruption^51,54^. In these dry-skin conditions, abnormal terminal differentiation of keratinocytes induces the disorder of functional stratum corneum formation that contributes to the skin barrier^49,51^. The decrease in wrinkles by dietary CAEP might be associated with an improvement in the skin barrier function because dryness influences the skin wrinkles.

Involucrin is present in the granular and upper spinous layers, and earlier clinical reports showed reduced involucrin expression, while premature expression after barrier disruption was observed in lower spinous layer^50,55^. In this study, the relatively low staining intensity of involucrin tended to be more broadly in the epidermis of HR-AD group compared to the other groups. Since involucrin participates in the maintenance of rigid cornified envelope, involucrin level might contribute to improvement in the skin barrier function^13^. Additionally, Jensen et al. suggested that decreased involucrin expression may cause a reduction in ω-hydroxyceramide levels in atopic dermatitis (AD) by failing to provide enough substrate for the binding of ceramides^50^. The immune-stained involucrin in the stratum corneum of CAEP group appeared to be more intense than in other groups, but it was not remarkable. The formation of stratum corneum is related to the upward migration and terminal differentiation of the keratinocytes in the epidermis ^13,50,55^. To elucidate the effects of dietary sphingolipids, further histological evaluation in detail is required. On the other hand, filaggrin expression was similar among CAEP, GluCer, and control groups. Filaggrin is a specific epidermal protein which is the precursor of the natural moisturizing factors and involved in the stratum corneum hydration^56^. In the dry-skin, filaggrin expression is downregulated^57^; however, filaggrin knock-down did not affect lipid composition of stratum corneum^58^. Hence, suggesting that a shortage of filaggrin participates in dry-skin, although filaggrin knock-down alone does not necessarily affect the barrier function^58^. Furthermore, Danso et al. reported that ELOVL1 and 6 expressions in the AD patients with filaggrin mutations were comparable to those in the wild-type AD patients^59^. Therefore, the effect of dietary CAEP on skin barrier function might not involve filaggrin expression changes.

Earlier reports showed a correlation between dry-skin and decrease in covalently bound ω-hydroxyceramide levels^16^. Covalently bound ω-hydroxyceramides are one of the major components of the cornified lipid envelope^14,47,60^. The impairment of covalently bound ω-hydroxyceramides production pathway causes ichthyosis^47,60^. Previous reports mentioned that covalently bound ω-hydroxyceramides are thought to play an important role in stabilizing lamellar structure as compared with other species^11,61^. In this study, administration of CAEP and GluCer increased epidermal covalently bound ω-hydroxyceramide levels and its synthesis-related gene expression in the skin compared to the control group. We found major covalently bound ω-hydroxyceramides in mice, although the minor molecule species (dihydrosphingosine-type, 4,14-sphingadiene-type and sphingosine-type involving odd-numbered carbon chain fatty acid) indicated by Kawana et al.^62^ were not detected. Dietary CAEP and GluCer intake effectively restore the skin condition in this mouse model, because ingestion of CAEP and GluCer normalizes the levels of epidermal ω-hydroxyceramide in mice compared to normal, hairless mice (data not shown). Interestingly, dietary CAEP and GluCer increased levels of covalently bound ω-hydroxyceramides having unsaturated fatty acids than those having saturated fatty acids. Previous studies reported that covalently bound ω-hydroxyceramides containing unsaturated fatty acid were less sensitive to aging, seasonal variation, and AD-like damage compared to those containing saturated fatty acid^16,61^. The presence of an unsaturated acyl chain is necessary for the formation of the lipid lamellar structure^63^. Importance of covalently bound ω-hydroxyceramides containing unsaturated fatty acid in maintenance and strengthening of epidermal lamellar structures are shown earlier^61^. In other previous reports, changes in not only covalently bound ω-hydroxyceramides but also epidermal ceramide profile or other lipids content are associated with skin barrier function^6,10^. Although this study evaluated ω-hydroxyceramides which are significantly associated with dry skin as the first step for elucidation of the effects of dietary CAEP on skin barrier, further studies to evaluate epidermal lipids profiles are needed to elucidate the more detailed mechanism of the effects. Macheleidt et al. reported that epidermal covalently bound ω-hydroxyceramides decreased in atopic dermatitis skin. In earlier reports, it has been shown that sphingomyelin rich diet and dietary GluCer exerted anti-inflammatory effects in the dry-skin hairless mouse model and a mouse model of oxazolone-induced chronic irritant contact dermatitis (ICD), respectively^16,64^. Therefore, dietary CAEP and GluCer may exert the improving effect on skin barrier function which may be contributed by covalently bound ω-hydroxyceramides synthesis and anti-inflammatory effects also in atopic dermatitis model mice, while this study has not focused on atopic dermatitis cytokines.

In this study, we showed an increase in the level of covalently bound ω-hydroxyceramide in the epidermis by dietary CAEP and GluCer comparable to milk sphingomyelin as reported earlier^16^. Fatty acids are elongated by the enzymes (ELOVL1-7) which have substrate specificities after conversion to acyl CoA^65–67^. In this study, dietary CAEP increased mRNA expression of ELOVL4, involved in the elongation of ultra-long-chain fatty acids (C26 <), highly expressed in the skin, and essential for skin barrier formation^68–71^. In ELOVL4 mutant mice, perinatal death by skin barrier disruption was caused by the deficiency of ultra-long-chain ceramides and acylceramides^72,73^. On the other hand, dietary GluCer upregulated mRNA expression of ELOVL1 in this study. ELOVL1 is involved in the elongation of very-long-chain fatty acids (especially C20-26) ^74^. Reports show reduced ELOVL1 mRNA levels in AD lesioned skin^59^. Sassa et al. showed early death in ELOVL1 knockout mice due to epidermal barrier defects^75^. Additionally, since C24 < very-long-chain acyl CoA is a substrate for ELOVL4, the upregulation of ELOVL1 might facilitate ultra-long-chain fatty acid synthesis^67^.

We also showed that dietary CAEP upregulated CERS2 and 3 mRNA expression compared to HR-AD and control groups. CERS3 synthesizes ultra-long-chain ceramide (C26 <), and its deficiency indicates skin barrier defect^60,77^. CERS2 synthesizes very-long-chain ceramides (C20 <)^77,78^. Moreover, Jennemann et al. reported that CERS3 uses ultra- but also very-long-chain acyl-CoAs as substrates. Thus, increased expression of ELOVL1 and CERS3 mRNA by dietary GluCer might also contribute to very-long-chain ceramide synthesis.

PNPLA1 plays an essential role in skin barrier function by catalysing the transacylation of the linoleic acid to ultra-long-chain ω-hydroxyceramide for acylceramide production^15,43,48^. Hydrolysis of the ester bond of oxidized linoleic acid residue of acylceramide produce ω-hydroxyceramide^60^. Covalently bound ω-hydroxyceramide is formed by crosslinking the exposed ω-OH groups of ω-hydroxyceramide with cornified envelope protein^60^. Thus, acylceramide is a precursor of covalently bound ω-hydroxyceramide. The enhancement of PNPLA1 mRNA expression by dietary CAEP shown in this study could contribute to improved efficacy of dietary CAEP on skin barrier function. On the other hand, TEWL and wrinkles were improved at day 3, so the expression of mRNAs may increase before the day 3.

An earlier report showed that each sphingoid base such as sphinganine (d18:0), d18:1 and 4,8-sphingadienine (d18:2) similarly enhanced mRNA expression of CERS2-4 in human foreskin keratinocytes^26^. Sphingatrienine (d18:3) and d19:3, purified from sea star, enhanced the de novo ceramide synthesis and mRNA expression of CERSs and ELOVLs in undifferentiated keratinocytes^79^. Thus, sphingoid bases derived from CAEP may have a similar effect on skin barrier function. Further investigation is needed to elucidate whether dietary CAEP enhances de novo synthesis of sphingoid bases which can contribute to the improving effect on skin barrier function. Additionally, sphingoid bases (d18:2 and t18:1 (4-hydroxy-8-sphinganine)) facilitated the expression of PPARβ/δ and PPARγ mRNA and acted as a ligand for PPARγ^80^. Recent studies have shown that activators of PPARα, PPARβ/δ, and PPARγ improved skin barrier function due to increased expression of epidermal ceramide synthesis-related genes and regulation of keratinocyte differentiation^81–83^. Thus, sphingoid bases might induce ceramide synthesis via PPAR activation.

Due to low dietary absorption of sphingoid bases, as shown earlier^42^, likely, sphingoid bases from dietary sources are hardly reutilized in the skin. However, even a modest delivery of sphingoid bases from dietary CAEP to skin might affect the ceramide synthesis because a low dose of sphingoid bases upregulated CERSs mRNA expression in keratinocytes^26^. CAEP used in this experiment is composed not only d18:1, which is a principal sphingoid base in mammals, but also unique sphingoid bases d19:3 and d16:1. d19:3 and d16:1 from CAEP were undetectable and d18:1 did not increase in the epidermis after CAEP administration in this study (data not shown). Therefore, suggesting that sphingoid bases derived from dietary sphingolipids are hardly reutilized to form the sphingolipids in the epidermis; however, they or their metabolites might play a role as triggers or signalling molecules in epidermal ceramide synthesis.

In conclusion, the present study shows a novel role of dietary CAEP on the improvement of skin barrier function and its involvement in the synthesis of epidermal covalently bound ω-hydroxyceramide constituted by ultra-long-chain ceramides. Moreover, the upregulation of ultra- and very-long-chain ceramide synthesis by dietary CAEP could contribute to the cornified lipid envelope formation. These effects of dietary CAEP were comparable to the dietary GluCer. The potential of dietary CAEP might be due to its easy digestion and absorption compared to other sphingolipids as described earlier^41,42^. Sphingoid base composition of maize GluCer was 66.1% d18:2, 13.6% t18:1, and others as described previously^29^. Structural difference among sphingolipids may affect their degradation and absorption. The findings in this study provide novel insights into the efficacy of dietary marine sphingophosphonolipid.

## Materials and methods

### Preparation of sphingolipids

CAEP was purified from crude lipids extracted from the skin of jumbo flying squid, *Dosidicus gigas*, kindly donated by Dr. Saito (Ishikawa Prefectural University, Japan) using previously described methods^41^. Constituent sphingoid base of the CAEP (purity 98%) were 41.4% d19:3, 28.6% d16:1, 12.9% d18:1, with smaller fractions of others as shown previously^41^. GluCer prepared from maize was kindly donated by Nippon Flour Mills Co. Ltd. (Atsugi, Japan). The purity of the GluCer was 92%.

### Animals and diets

Female hairless mice (Hos: HR-1, 4-week-old) were purchased from Hoshino Laboratory Animals, Inc (Ibaragi, Japan) and maintained following the Guide for the Care and Use of Laboratory Animals (Animal Care Committee, Kyoto University). Approval for all protocols used in this study was from the Kyoto University animal committee (no. 27–37). The mice were individually housed in plastic cages at 24 ± 1 °C with a 12-h light/dark cycle and free access to diet and distilled water. All mice were fed MF standard chow (Oriental Yeast, Tokyo, Japan) for a week, acclimated before the start of the experiments, and were subjected to the experimental protocols as described earlier^26^. For developing the dry-skin condition (i.e., perturbations in the skin barrier), mice were fed magnesium-deficient diet (HR-AD chow, Nosan Corp., Yokohama, Japan) for 11 weeks^47,84^. Subsequently, mice were randomly divided into four groups. The mice sacrificed immediately after the HR-AD feeding period were designated as HR-AD group (n = 5). After the HR-AD feeding period, mice in the control group were fed AIN-93G for seven days (n = 5). Mice in the CAEP and GluCer group received the AIN-93G diet containing 0.1% CAEP and 0.1% GluCer, respectively (n = 6 in each group) (Table S2). Body weights and dietary intake of each mouse were measured daily. At the end of each treatment, mice were sacrificed under isoflurane anaesthesia, and the dorsal skin specimens collected immediately. Pieces of dorsal skin were fixed in 10% neutral buffered formalin solution for morphological analysis. To analyse the mRNA expression, part of the skin specimens was stored in RNAlater (Qiagen, Valencia, CA) at − 80 °C until use. The skin specimens for lipid analysis were frozen immediately at − 80 °C.

### Measurement of skin barrier functions

TEWL and the dorsal skin hydration index of the mice were measured on day-0, -3, and -6 after switching the HR-AD diet to the experimental diets. The measurements of TEWL and the hydration index by Tewameter TM 300 and Corneometer CM 825 (Courage Khazaka electronic GmbH, Cologne, Germany), respectively, were continued until the TEWL recovered to the normal level (below 10 g/m^2^/h).

### Evaluation of the wrinkles in the skin

On day 0, 3, and 6 of the recovery treatment, the skin surface replicas were collected from the dorsal skin, using a silicone product (Asch Japan Co., Ltd, Hachioji, Japan) under isoflurane anaesthesia. For evaluating the wrinkling degree, parameters (number of wrinkles, the average depth of wrinkles, wrinkle area ratio, and wrinkle volume ratio) of the skin replica were measured using skin wrinkle analysis software (Asch Japan Co., Ltd)^52,53^.

### Histological analysis of epidermis

For evaluating the morphological changes, pieces of dorsal skin were sectioned and stained with haematoxylin and eosin (H&E) by Biopathology Institute (Oita, Japan). Immunohistochemical staining with antibody against involucrin and filaggrin was performed for typical two animals in each group at Biopathology Institute. The thickness of the epidermis and the area of immune-stained involucrin were measured using a Biorevo all-in-one microscope (BZ-9000, Keyence Co., Osaka, Japan) and Image J software (Wayne Rasband, National Institutes of Health, Bethesda, Maryland, USA), respectively. The average value of 10 random determinations was considered as the representative value for the individual animal.

### HPLC analysis of covalently bound ceramides in the epidermis

Covalently bound ceramides were extracted with a slight modification of earlier methods^12,16^. The skin epidermis was separated from the dermis at the basement membrane by overnight incubation at 4 °C with 2.5 U/mL Dispase II (neutral protease, grade II, Roche Diagnostics GmbH Mannheim, Germany) in Hanks' balanced salt solution + (HBSS( +), Nacalai Tesque). After removal of the epidermal lipids using chloroform/methanol (2:1, v/v), ω-hydroxyceramides bound to the stratum corneum by ester bonds were released by overnight incubation in 1 M KOH in 95% methanol at room temperature. Subsequent extraction of ω-hydroxyceramides was with chloroform/methanol (2:1, v/v) after neutralization with acetic acid. The protein level in the residue quantified using a DC Protein Assay kit (Bio-Rad Laboratories, CA, USA). Analysis of ω-hydroxyceramides was done using HPLC system coupled with an ion trap time-of-flight mass-spectrometer (LCMS-IT-TOF; Shimadzu Co., Kyoto, Japan), equipped with atmospheric pressure chemical ionisation (APCI) or electrospray ionisation (ESI) interface (Shimadzu) using an earlier method with some modifications^42^. Mobile phase A consisted of 1 mM ammonium acetate in methanol and 2 mM ammonium acetate in water (80:20, v/v). Mobile phase B consisted of 1 mM ammonium acetate in methanol and 2 mM ammonium acetate in water (99:1, v/v). The TSKgel ODS-100Z column (2.0 mm × 50 mm, i.d., 3 μm, Tosoh, Tokyo, Japan) was eluted using the following binary gradients: 0–5 min, 50–100% B; 5–35 min, 100% B; 35–40 min, 100–50% B. The MS (range, m/z 400 to 1,200) and MS/MS (range, m/z 200 to 350) spectra were acquired in the positive scan mode. For structural analysis of ω-hydroxyceramides, [M + H – H_2_O]^+^ was used to obtain product ions by MS/MS analysis following earlier method^36,42^. In Table S1, typical signals of d17:1 [M + H – 2H_2_O]^+^ (m/z 250.3) and d18:1 [M + H – 2H_2_O]^+^ (m/z 264.3), characteristic sphingoid bases present in the mouse skin^54^ were observed as product ions using auto MS/MS detection mode^16^. Pairs of structurally-specific product ions of sphingoid bases and their precursor-ions were used to identify ω-hydroxyceramide molecules. The precursor-ions as [M + H – H_2_O]^+^ were used to semi-quantify each molecule of ω-hydroxyceramide by LCMS-2010 eV (Shimadzu) equipped with APCI probe. The analytical conditions were as described before with some modifications^41^. HPLC conditions were like that of LCMS-IT-TOF analysis described above. The relative peak area of ω-hydroxyceramide was calculated using an authentic standard (N-*omega*-hydroxy-C30:0-_D_-*erythro*-ceramide, Matreya LLC, Inc., Pleasant Gap, PA, USA).

### RNA preparation and real-time qRT-PCR

Total RNA extraction, cDNA synthesis, and real-time quantitative qPCR were performed as described before with some modifications^26,53^. Total RNA was extracted from the skin samples in RNAlater by using Sepasol reagent (Nacalai Tesque) following the manufacturer's instructions. cDNAs were synthesized using SuperScript RNase II reverse transcriptase (Invitrogen, CA, USA) with random hexamers. For RT-PCR, 3 μL diluted cDNA was mixed with 7 μL iQ SYBR Green Supermix (Bio-Rad Laboratories, CA, USA) containing 2 μL PCR primer (5 μM, primer sequences are shown in Table S3). Real-time qRT-PCR performed by using a DNA Engine Option system (Bio-Rad Laboratories). The thermal cycling conditions were 3 min at 95 °C for 1 cycle, followed by amplification for 40 cycles with melting for 15 s at 95 °C, and annealing and extension for 30 s at 60 °C. The expression level of each gene was normalized using β-actin (ACTB) mRNA as an internal control.

### Statistical analysis

Data are reported as means ± standard errors. Statistical analysis was by using Stat View software (SAS Institute, NC, USA). Two-way repeated ANOVA analysed body weight and food intake. Analysis of other measurements was by one-way ANOVA and Tukey–Kramer tests as a post hoc test. Data were considered statistically significant with *P*-values < 0.05.

## Supplementary information

Supplementary information
