# Supplementary material for: Dietary ceramide 2-aminoethylphosphonate, a marine sphingophosphonolipid, improves skin barrier function in hairless mice
Source: Sci Rep. 2020 Aug 17;10:13891. doi: 10.1038/s41598-020-70888-0 (PMC7431532; doi:10.1038/s41598-020-70888-0)
Supplement: Supplementary file 1 — Supplementary information [file 41598_2020_70888_MOESM1_ESM.docx]

**Dietary ceramide 2-aminoethylphosphonate, a marine sphingophosphonolipid, improves skin barrier function in hairless mice**

Nami Tomonaga^1^, Yuki Manabe^1^, Kazuhiko Aida^2^ & Tatsuya Sugawara^1*^

^1.^ Laboratory of Technology of Marine Bioproducts, Division of Applied Biosciences, Graduate School of Agriculture, Kyoto University, Kitashirakawaoiwakecho, Sakyo-ku, Kyoto 606-8502, Japan

^2.^ Innovation Center, Nippon Flour Mills Co., Ltd., 5-1-3 Midorigaoka, Atsugi, Kanagawa, 243-0041, Japan

*Correspondence and requests for materials should be addressed to T.S. (email: [sugawara@kais.kyoto-u.ac.jp](mailto:sugawara@kais.kyoto-u.ac.jp))

**Supplementary Table S1**

Typical covalently bound ω-hydroxyceramide molecular species in mice epidermis fed CAEP diet analysed by MS/MS

| **Molecule** | **Molecular**  **formula** | **Precursor ion**  ***m/z*** | **Product ion**  ***m/z*** | **Peak area** | **Retention time**  **(min)** |
| --- | --- | --- | --- | --- | --- |
| d17:1/C32:1h | C_49_H_95_NO_4_ | 744.7 | 250.3 | 45,418,699 | 12.7 |
| d18:1/C30:0h | C_48_H_95_NO_4_ | 732.7 | 264.3 | 15,581,484 | 13.6 |
| d18:1/C32:1h | C_50_H_97_NO_4_ | 758.7 | 264.3 | 95,415,579 | 14.2 |
| d17:1/C32:0h | C_49_H_97_NO_4_ | 746.7 | 250.3 | 44,153,407 | 15.3 |
| d17:1/C34:1h | C_51_H_99_NO_4_ | 772.8 | 250.3 | 123,835,001 | 15.8 |
| d18:1C32:0h | C_50_H_99_NO_4_ | 760.8 | 264.3 | 43,109,580 | 17.0 |
| d18:1/C34:1h | C_52_H_101_NO_4_ | 786.8 | 264.3 | 164,652,959 | 17.8 |
| d17:1/C36:1h | C_53_H_103_NO_4_ | 800.8 | 250.3 | 21,485,808 | 19.8 |
| d18:1/C34:0h | C_52_H_103_NO_4_ | 788.8 | 264.3 | 2,323,941 | 21.8 |
| d18:1/C36:1h | C_54_H_105_NO_4_ | 814.8 | 264.3 | 12,665,686 | 22.6 |

**Supplementary Table S2**

Experimental diet compositions used in dry-skin models (g/100 g diet)

| **Ingredient (g)** | **Control**  **(AIN-93G)** | **0.1%**  **CAEP** | **0.1%**  **GluCer** |
| --- | --- | --- | --- |
| Cornstarch | 39.7486 | 39.7486 | 39.7486 |
| Casein | 20.0 | 20.0 | 20.0 |
| Dextrinized corn starch | 13.2 | 13.2 | 13.2 |
| Sucrose | 10.0 | 10.0 | 10.0 |
| Soybean oil | 7.0 | 6.9 | 6.9 |
| Cellulose powder | 5.0 | 5.0 | 5.0 |
| AIN-93G mineral | 3.5 | 3.5 | 3.5 |
| AIN-93 vitamin | 1.0 | 1.0 | 1.0 |
| L-Cystine | 0.3 | 0.3 | 0.3 |
| Choline bitartrate | 0.25 | 0.25 | 0.25 |
| Buthyl hydroxy toluene | 0.0014 | 0.0014 | 0.0014 |
| CAEP |  | 0.1 |  |
| GluCer |  |  | 0.1 |
| Total | 100 | 100 | 100 |

**Supplementary Table S3**

GenBank accession numbers and primer sequences used in real-time qRT-PCR experiments

| **Gene** | **GenBank accession no.** | **Forward (from 5' to 3')** | **Reverse (from 5' to 3')** |
| --- | --- | --- | --- |
| CERS2 | NM_029789.2 | CCTCTGCTTCTCCTGGTTTG | GGCGAACACAATGAAGAGGT |
| CERS3 | NM_001164201.1 | ATCTCGAGCCCTTCTTCTCC | CTCTGTCTCTTTGCCCTTGG |
| ELOVL1 | NM_019422.3 | TCCCTTTGAACCCTTCACTG | ACCCCATCAGAGGGTAGCTT |
| ELOVL4 | NM_148941.2 | ACTATGGGCTGACTGCGTTC | GGGCAGTCGGTGTAGAGAGA |
| PNPLA1 | NM_001034885.3 | GTCTCTTGGTCGCTGACGTT | GGCTGGAGATTGAGGCTTAG |
| ACTB | NM_007393.5 | CCTCTATGCCAACACAGTGC | GTACTTGCGCTCAGGAGGAG |
